# Can digital economy foster synergistic increases in green innovation and corporate value? Evidence from China

**DOI:** 10.1371/journal.pone.0304625

**Published:** 2024-06-13

**Authors:** Guomin Song, Fengyan Wang, Feng Dong

**Affiliations:** 1 School of Economics and Management, China University of Mining and Technology, Xuzhou, China; 2 School of Accounting, Shandong Women’s University, Jinan, China; Universiti Teknologi MARA, MALAYSIA

## Abstract

The rapid evolution of the digital economy has significantly accelerated progress towards achieving green and sustainable processes, particularly in the field of green production. While existing research has delved into the effects of the digital economy on Green Innovation (GI) and the consequences of digital transformation on Corporate Value (CV), there remains a notable gap in the literature regarding the potential for synergistic enhancements in firms’ GI&CV through the ongoing digital revolution. This study utilizes an evolutionary game model and employs system dynamics methods to simulate the dynamic evolution trajectory of the influence of the digital economy on the synergy between GI&CV. Subsequently, it empirically assesses the interconnected synergies between GI&CV using a dataset comprising information from Chinese listed firms spanning from 2011 to 2020, examining the impact of the digital economy on these synergies. Moreover, the study delves into the analysis of the transmission mechanism and conducts an extended investigation to further explore this phenomenon. The findings of this paper including: (1) The digital economy acts as a driving force behind the synergistic enhancement of firm GI&CV. Moreover, this effect is further augmented by governmental environmental regulation and green subsidy policies. (2) Drawing upon the information asymmetry theory and the resource-based theory, the regional marketization level and firms’ digital transformation play intermediary roles. (3) The heterogeneity test indicate that firms situated in eastern regions and those classified as non-heavily polluted benefitted to a greater extent. This study sheds light on the incentive implications of digital economy for the synergistic upgrading of GI&CV, thereby extending the breadth of study on the consequences of digital economy. Moreover, it offers actionable suggestions for enterprises to leverage digital economy development towards achieving a synergistic improvement of GI&CV.

## 1. Introduction

As a new economic paradigm typically characterized by innovation and spillover, the digital economy effectively enhances the developmental resilience of the regional economy and deeply empowers the real economy [1], becoming an important engine to promote the conversion of firms from factor-driven to innovation-driven mode and to enhance the value of the company. The effects of digital technology on the economy are reflected not only in a single industry, but throughout the entire value chain [2], with significant implications for the entire economic system. The digital economy brings great innovation potential for enterprise development [3], and also promotes the exploitation of emerging green industries [4]. For example, the development of new energy, new materials, green environmental protection equipment and other fields make economic activities more efficient, intelligent and sustainable. The study of digital economy can help us recognize and explore these green innovation potentials, thus promoting the two-way enhancement of GI&CV.

Based on the driving force of GI in the digital economy, numerous studies have been conducted by scholars regarding the digital economy and GI. The majority of these studies have reached the consensus that the digital economy generates positive externalities and fosters sustainability [5–7]. Other scholars posit that the digital economy significantly contributes to the promotion of enterprise value creation [8, 9] Primarily focusing on individual perspectives, these studies have investigated the influence of the digital economy on GI either the impact of digital transformation on CV. However, there remains a dearth of research exploring the potential synergistic enhancement of firms’ GI&CV though the digital economy revolution. Additionally, the underlying mechanisms that facilitate the influence between these two factors have not been sufficiently explored. Hence, this paper seeks to address several key inquiries: How will the digital economy affect the GI&CV synergy? What is the underlying mechanism that dictates this effect? To what extent do additional variables strengthen or moderate these effects? Furthermore, are there discernible discrepancies across different regions and industries? By providing answers to these aforementioned questions, this research aims to elucidate the operating mechanisms and consequential effects resulting from the digital economy on the synergistic relationship of GI&CV. Furthermore, this research sheds light on the incentive implications of digital economy for the synergistic upgrading of GI&CV, offers actionable suggestions for enterprises to leverage digital economy development towards achieving a synergistic improvement of GI&CV, thereby extending the breadth of study on the consequences of digital economy. Simultaneously, these findings will empower governments to devise intricate strategies for digital economic development that effectively nurture the synergistic improvement of green innovation and corporate value within enterprises.

To comprehensively delve into the intricate relationship among digital economy development and the synergy of GI&CV, as well as their associated influence mechanisms, this study employs a capacitive coupling coefficient model to measure the synergy of GI&CV. Empirical analysis is conducted using data from Chinese listed companies spanning the period from 2011 to 2020. The results reveal that digital economy development stimulates corporate GI and bolsters CV either, thereby accomplishing a symbiotic relationship and coupling effect between these two factors. From the perspective of function channels, the regional marketization level and the digital transformation act as mediating factors between digital economy and the GI&CV synergy based on the information asymmetry theory and resource based theory. The comprehensive analysis reveals that government-enforced environmental regulations and green subsidies amplify the positive influence of digital economy on the synergy of GI&CV. Heterogeneity analysis uncovers several key findings. Firstly, variations in sensitivity are observed across different economic regions: digital economy development exhibits greater sensitivity to the impact of the synergy of GI&CV among firms in the eastern region, while the enhancement of synergies for firms in the central and northeastern regions is more pronounced. Conversely, digital economy development does not contribute to the synergy of GI&CV in the western region. Secondly, digital economy development proves more instrumental in promoting the synergy of GI&CV for non-heavy pollution firms. This study not only expands upon the existing research regarding the dialectical relationship between enterprise GI&CV but also furnishes valuable insights on leveraging digital economy development to enhance the synergy between enterprise GI&CV. These discoveries provide a critical benchmark for effectively fostering the synergistic improvement of GI&CV within organizations.

Accordingly, this paper delivers three primary marginal contributions. Firstly, existing research predominantly concentrates on the vertical impact of the digital economy on GI or CV, while giving less attention to the analysis of potential horizontal coupling or synergy effects. The intricate relationship between green innovation and enterprise value underscores the necessity of acknowledging their interconnectivity. While they function as distinct systems, their synergy is undeniable. Realizing optimal outcomes demands a comprehensive approach that holistically considers their interplay. This article endeavors to quantify this interdependence through the development of a capacity coupling coefficient model. The findings offer valuable insights for firms on strategies to enhance CV while implementing GI.

Secondly, previous research primarily relies on empirical evidence, overlooking a game-theoretic perspective to investigate the mechanism whereby the digital economy influences GI or CV. In this paper, we provide a detailed account of the impact of government investment in digital economy construction on the synergy between GI&CV. To achieve this, we construct an evolutionary game model and employ system dynamics simulation analysis.

Thirdly, previous research has primarily investigated the influence of the digital economy on a singular dimension of GI or CV, neglecting to consider the potential enhancement of GI&CV synergy resulting from the digital economy. To address this gap, our research investigates the driving force behind the digital economy on firms’ synergies of GI&CV. Additionally, we unveil the effect mechanism of this driving force from two viewpoints: synergistic governance theory and dynamic capability theory. By delving into the nuanced dynamics of enterprise green innovation’s economic implications, this research not only provides deeper insights but also furnishes policymakers with actionable recommendations. These insights are crucial for optimizing digital economy policies, thereby facilitating the seamless integration of green innovation initiatives and value creation within enterprises.

The structure of this paper is outlined as follows: Section 2 presents the literature review, Section 3 conducts an evolutionary game analysis, Section 4 performs metrological inspections, Section 5 engages in a comprehensive discussion, and Section 6 summarizes the findings and proposes countermeasures.

## 2. Literature review

### 2.1 The impact of digital economy on enterprise green innovation

With the global promotion of the concept of green sustainable development, the relationship between the digital economy and green innovation has garnered significant academic attention in recent years. In the latest wave of technological revolution and industrial transformation, the role of the digital economy in driving green innovation has been extensively explored and validated across various domains. The advancement of the digital economy has led to a profound impact on the economy by accelerating the commodification and commercialization of data. This has reshaped the traditional structure of factor inputs in the economy, facilitating the digital transformation of production factors [10, 11]. Consequently, it has improved enterprise production efficiency, optimized resource allocation, and reduced resource consumption [12, 13]. These changes have not only facilitated green innovation but have also contributed to enhancing the value creation process within enterprises. Moreover, the rapid development of artificial intelligence and big data technologies has further spurred the digital transformation of enterprise manufacturing systems and supply chains. This has bolstered the fusion of environmentally-friendly business practices with lean practices, enabling enterprises to enhance their green innovation performance [14, 15].

Several researchers have examined how the digital economy facilitates green innovation. By leveraging economies of scale, economies of scope, and other factors, the digital economy is capable of reducing the cost of information communication [16, 17] and enhancing production efficiency [18, 19], thereby stimulating GI [20, 21]. Externally, the advancement of the digital economy can bolster companies’ green innovation through various means, such as boosting economic openness, optimizing industrial structure, expanding market potential, increasing media visibility, and reducing environmental uncertainty [6, 14, 22, 23]. Internally, digital progress can drive green innovation by refining human capital structures, easing financial constraints, enhancing internal controls, strengthening human capital, and fostering the enhancement of the enterprise value chain [5, 24, 25]. For instance, Liu (2022) demonstrated in their study that the digital economy substantially enhances enterprises’ level of substantive GI by improving internal control quality and increasing long-term investment [26]. Furthermore, Zhang (2023), through an analysis of data from listed manufacturing enterprises, concluded that digital transformation significantly contributes to enterprises’ GI [27].

### 2.2 The impact of digital economy on enterprise value creation

The exploration of the correlation between digital technology revolution or digital transformation and corporate value is another topical issue in the field of digital economy research. The rise of the digital economy has significantly disrupted traditional value creation models within enterprises through a transformation of their production methods and service delivery mechanisms [28–31]. This shift has been accompanied by the emergence of digital platforms and ecosystems, facilitated by the digital economy, which present businesses with enhanced commercial prospects and innovative opportunities. These platforms and ecosystems not only foster strategic alignment within organizations but also catalyze a fundamental evolution in value creation processes for enterprises, as highlighted by recent studies [9, 32]. Digital technology can break organizational or industrial boundaries [33], broaden the path of enterprise value creation, and promote digital business model transformation and innovation [34–36]. Moreover, the expansion of the digital economy and the adoption of digital technologies have empowered companies to gain a comprehensive understanding of customer preferences, deliver tailored products and services, establish novel distribution channels, enhance user engagement, and cultivate brand loyalty. Consequently, these initiatives contribute to a significant increase in enterprise value, as evidenced in recent research [37]. Furthermore, the progression of digital technologies equips organizations with improved capabilities to effectively capture, analyze, and leverage data, thereby driving data-informed decision-making processes, enhancing awareness of market dynamics and customer requirements, facilitating strategic planning, stimulating digital transformation, optimizing operational workflows, and ultimately paving the way for sustained enterprise growth and a competitive edge in the long run [8, 38]. For instance, digital supply chain management has been shown to diminish inventory and transportation expenses [39], while enhancing supply chain resilience and diversification [40]. Additionally, data analytics can provide firms with profound insights into market responses and customer preferences, facilitating informed strategic management decisions [41], thereby potentially augmenting enterprise market value [42].

## 3. Game analysis

### 3.1 Basic assumptions of the game

Drawing upon the inherent logical relationship between the government and the firm’ behavior, the ensuing fundamental assumptions are posited as follows:

Assumption 1: The entities engaged in the game are the government (denoted as ’*g*’) and the firm (denoted as ’*e*’). Both parties exhibit bounded rationality, and their behaviors are mutually influential.
Assumption 2: The initial utility of the government, denoted as *W*_*g*_, is influenced by the implementation of high-level digital economy policies. This implementation necessitates an augmented investment in digital economy infrastructure, denoted as *C*_*g*_. Simultaneously, the government can obtain digital returns, denoted as *R*_*g*_. Conversely, the government’s adoption of low-level digital economy policies triggers a reduction in both its investment in digital economy infrastructure, to *αC*_*g*_ (where 0 < *α* < 1), and digital revenue, to *βR*_*g*_ (where 0 < *β* < 1).
Assumption 3: The initial return of the firm is denoted as *W*_*e*_. When the firm chooses to synergize with GI&CV, it will undertake the synergy costs, denoted as *C*_*e*_, while also obtaining sustainable development benefits, denoted as *S*_*e*_. On the contrary, if the firm chooses not to synergize, there is no need to pay for synergy costs, but the enterprise cannot obtain sustainable development benefits either.
Assumption 4: When the government implements high-level digital economy policies, the firm will benefit from the resource opportunities created by the digital economy and the increased digital revenue, denoted as *R*_*e*_, and the government can obtain the tax revenue, denoted as *tR*_*e*_, brought by the increase in enterprise revenue. When the government implements low-level digital economy policies, the digital benefits accruing to firms decrease to *γR*_*e*_ (where 0<*γ*<1). Failure to pursue synergy renders firms unable to attain sustainable development benefits and digital advantages, thereby incurring environmental compliance costs denoted as *D*_*e*_.

### 3.2 Game payoff matrix and payoff expectation function

Based on the above game assumptions, the evolutionary game payoff matrix constructed is interpreted in Table 1.

**Table 1:** Benefits matrix for the government and the firm.

Entities of the game and strategies		The government	
		High-level digital economy(x)	Low-level digital economy(1-x)
The firm	GI&CV synergize (y)	*W*_*g*_ − *C*_*g*_ + *R*_*g*_ + *tR*_*e*_	*W*_*g*_ − *αC*_*g*_ + *βR*_*g*_ + *tγR*_*e*_
		*W*_*e*_ − *C*_*e*_ + *R*_*e*_ + *S*_*e*_	*W*_*e*_ − *C*_*e*_ + *γR*_*e*_ + *S*_*e*_
	GI&CV not synergize(1-y)	*W*_*g*_ − *C*_*g*_ + *R*_*g*_ + *D*_*e*_	*W*_*g*_ − *αC*_*g*_ + *βR*_*g*_ + *D*_*e*_
		*W*_*e*_ − *D*_*e*_	*W*_*e*_ − *D*_*e*_

According to Tables 3–5, the expected and average returns to the government can be obtained:

$$E_{g}^{1}=y\left(W_{g}-C_{g}+R_{g}+{tR}_{e}\right)+(1-y)\left(W_{g}-C_{g}+R_{g}+D_{e}\right)$$ (1)

$$E_{g}^{2}=y\left(W_{g}-{\alpha C}_{g}+{\beta R}_{g}+t{\gamma R}_{e}\right)+(1-y)\left(W_{g}-{\alpha C}_{g}+{\beta R}_{g}+D_{e}\right)$$ (2)

$$\bar{E_{g}}=xE_{g}^{1}+\left(1-x\right)E_{g}^{2}$$ (3)

According to Eqs (1)–(3), the equation for the replication dynamics of the government can be obtained as:

$${F\left(x\right)}^{*}=\frac{dx}{dt}=x{(E}_{f}^{1}-\bar{E_{f}})=x\left(1-x\right)\left[{(1-\beta )R}_{g}-\left(1-\alpha \right)C_{g}+ty(1-\gamma )R_{e}\right]$$ (4)

Similarly, the firm’s expected and average returns are known to be:

$$E_{e}^{1}=x\left(W_{e}-C_{e}+R_{e}+S_{e}\right)+(1-x)\left(W_{e}-C_{e}+{\gamma R}_{e}+S_{e}\right)$$ (5)

$$E_{e}^{2}=x\left(W_{e}-D_{e}\right)+(1-x)\left(W_{e}-D_{e}\right)$$ (6)

$$\bar{E_{e}}=yE_{e}^{1}+\left(1-y\right)E_{e}^{2}$$ (7)

Based on Eqs (5)–(7), the equation for the replication dynamics of firm e can be obtained as:

$$F\left(y\right)=\frac{dy}{dt}=y{(E}_{e}^{1}-\bar{E_{e}})=y\left(1-y\right)\left(D_{e}-C_{e}+S_{e}+{\gamma R}_{e}+x(1-\gamma )R_{e}\right)$$ (8)

### 3.3 Equilibrium and stability analysis

According to the stability equilibrium conditions of the Jacobian matrix, four combinations of values satisfying the equilibrium conditions are identified: (0, 0), (1, 0), (0, 1), and (1, 1). The evolutionary stabilization strategies of each entity are presented in Table 2.

**Table 2:** Evolutionary stabilization strategies.

Case	Equilibrium Condition	Equilibrium	trJ	deJ
(1)	*C*_*g*_ > (1 − *β*)*R*_*g*_/(1 − *α*) and *C*_*e*_ > *D*_*e*_ + *S*_*e*_ + *γR*_*e*_	ESS(0,0)	-	+
(2)	$0<C_{g}<\frac{\left(1-\beta \right)R_{g}}{1-\alpha }$ and *C*_*e*_ > *D*_*e*_ + *S*_*e*_ + *γR*_*e*_	ESS(1,0)	-	+
(3)	*C*_*g*_ > [(1 − *β*) *R*_*g*_ + *t*(1 − *γ*)*R*_*e*_]/(1 − *α*) and 0 < *C*_*e*_ < *D*_*e*_ + *S*_*e*_ + *γR*_*e*_	ESS(0,1)	-	+
(4)	${0<C}_{g}<\frac{\left[\left(1-\beta \right)R_{g}+{t\left(1-\gamma \right)R}_{e}\right]}{1-\alpha }$ and 0 < *C*_*e*_ < *D*_*e*_ + *S*_*e*_ + *R*_*e*_	ESS(1,1)	-	+

### 3.4 System dynamics simulation analysis

To provide a more intuitive explanation of the government’s role in GI&CV synergies, this paper utilizes MatlabR2022 to conduct a system dynamics simulation analysis.

**Fig 1:**
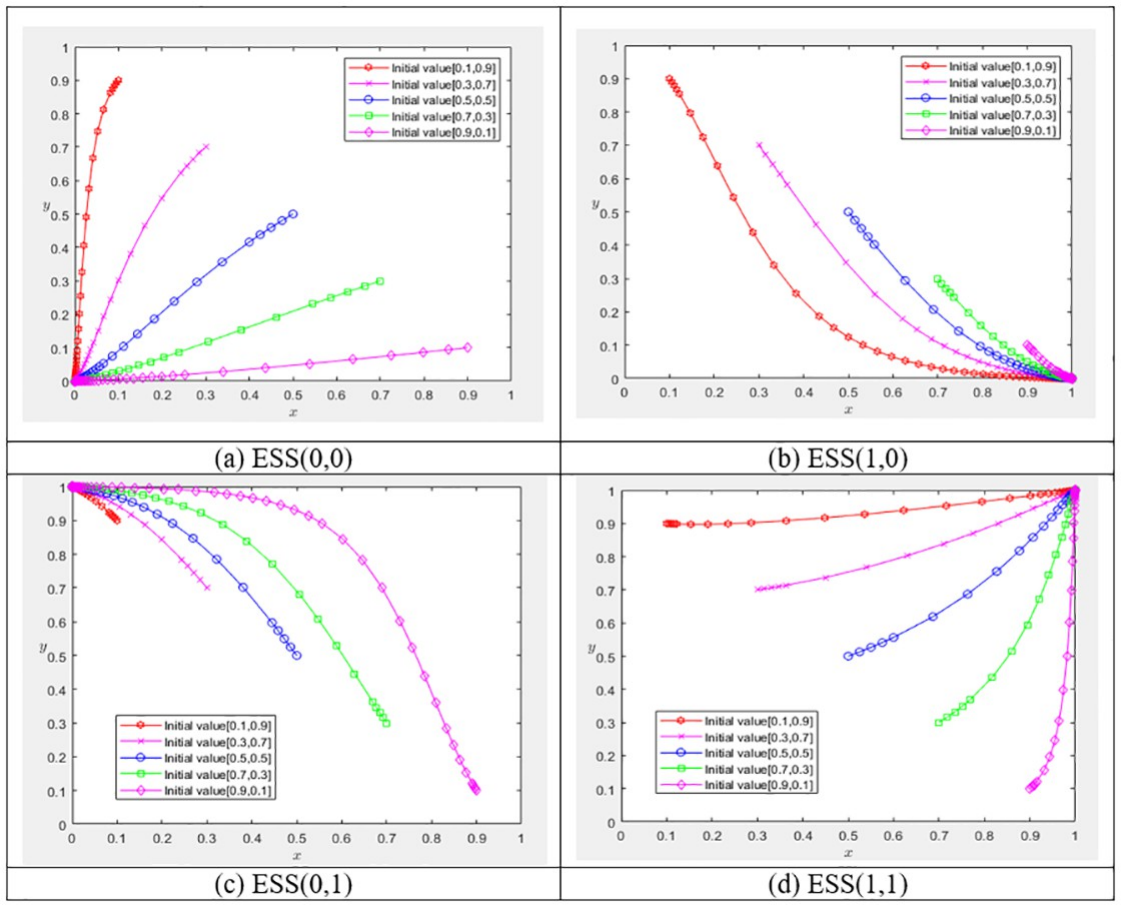
Evolution trajectory of the game between the government and the firm.

*(1) Simulation analysis*. When *C*_*g*_ > (1 − *β*)*R*_*g*_/(1 − *α*) and *C*_*e*_ > *D*_*e*_ + *S*_*e*_ + *γR*_*e*_, the variables are assigned as *C*_*g*_ = *30*, *R*_*g*_ = *10*, *C*_*e*_ = *40*, *D*_*e*_ = *10*, *R*_*e*_ = *20*, *S*_*e*_ = *10*, *α* = *0*.*3*, *β* = *0*.*4*, *γ* = *0*.*4*, *t* = *0*.*2*, and the evolution trajectory of each subject is shown in Fig 1(a). The government’s expenditure on implementing the digital economy surpasses the benefits gained from digitization, thus leading it to opt for a lower level of investment in the digital economy. Similarly, the synergy cost associated with firms’ GI&CV outweighs the cumulative benefits for the firm, consequently diminishing their inclination to pursue synergistic efforts. Hence, the firm opt not to synergize GI&CV.

When 0 < *C*_*g*_ < (1 − *β*)*R*_*g*_/(1 − *α*) and *C*_*e*_ > *D*_*e*_ + *S*_*e*_ + *γR*_*e*_, the variables are assigned *C*_*g*_ = *10*, *R*_*g*_ = *20*, *C*_*e*_ = *40*, *D*_*e*_ = *10*, *R*_*e*_ = *10*, *S*_*e*_ = *10*, *α* = *0*.*3*, *β* = *0*.*4*, *γ* = *0*.*4*, *t* = *0*.*2*, and the trajectory of each subject is shown in Fig 1(b). The government’s investment costs for digital economy infrastructure construction decline significantly, choosing to implement a high level of digital economy becomes the optimal strategy. Conversely, despite the persistent high synergy costs of GI&CV, the firm opt for separate GI&CV strategies without pursuing synergy.

When $C_{g}>\frac{\left(1-\beta \right)R_{g}+{t\left(1-\gamma \right)R}_{e}}{1-\alpha }\;$ and 0 < *C*_*e*_ < *D*_*e*_ + *S*_*e*_ + *γR*_*e*_, the variables are assigned *C*_*g*_ = *30*, *R*_*g*_ = *10*, *C*_*e*_ = *40*, *D*_*e*_ = *10*, *R*_*e*_ = *20*, *S*_*e*_ = *30*, *α* = *0*.*3*, *β* = *0*.*4*, *γ* = *0*.*4*, *t* = *0*.*2*, and the trajectory of each subject is shown in Fig 1(c). The investment cost associated with the government’s implementation of the digital economy exceeds its digital benefits, leading the government to opt for a lower level of investment. Conversely, the cost incurred by the firm for GI&CV synergy has notably declined, prompting the firm to choose collaboration.

Fig 1(d) shows the case (4), when ${0<C}_{g}<\frac{\left(1-\beta \right)R_{g}+{t\left(1-\gamma \right)R}_{e}}{1-\alpha }\;$ and 0 < *C*_*e*_ < *D*_*e*_ + *S*_*e*_ + *R*_*e*_, the variables are assigned *C*_*g*_ = *10*, *R*_*g*_ = *20*, *C*_*e*_ = *40*, *D*_*e*_ = *20*, *R*_*e*_ = *20*, *S*_*e*_ = *10*, *α* = *0*.*3*, *β* = *0*.*4*, *γ* = *0*.*4*, *t* = *0*.*2*. In contrast with ESS(0,1), the government’s investment in the digital economy is lower than the corresponding digital benefits, and the cost of synergizing GI&CV for the firm is significantly lower than the total synergistic benefits including other associated advantages. Consequently, the government opts for a high-level digital economy, while the firm choose to pursue synergy between GI&CV.

Based on the above analysis, the synergy of GI&CV is influenced by factors such as investment in digital economy construction, synergy costs, and digital benefits. To further examine how the variable of digital economy intensity affects the synergy of GI&CV, we carry out a sensitivity test to the digital economy intensity.

### 3.5 Sensitivity test of digital economy intensity

**Fig 2:**
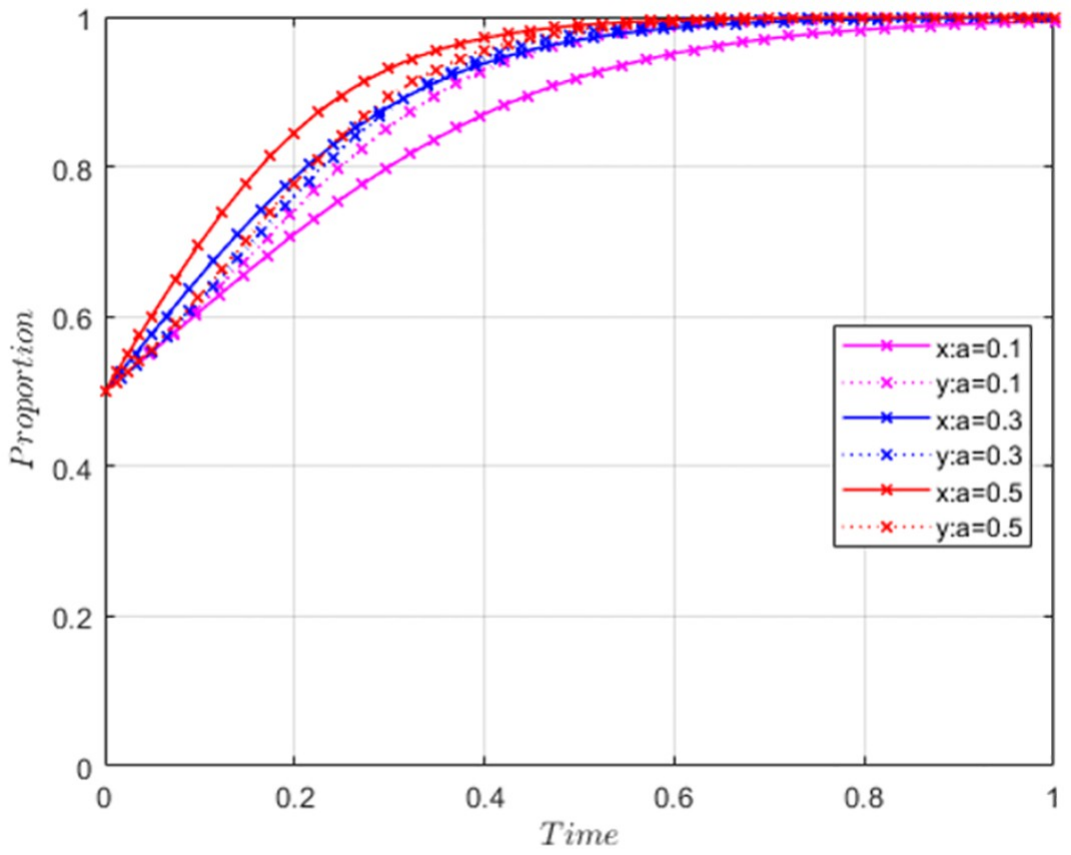
Sensitivity test of the digital economy intensity.

In this study, we introduce the coefficient α to represent the government’s increased investment in digital economy infrastructure. This coefficient reflects the level of intensity in the government’s efforts towards digital economy construction. Therefore, we employ the variable ’α’ as a substitute variable to measure the digital economy intensity for sensitivity analysis. Fig 2 presents the asymptotic stable evolution trajectory of the influence of varying digital economy intensity values on the synergy of GI&CV (represented by ’y’). This trajectory is observed under the evolutionary stability strategy applied by both the government and the firm, when the ESS is (1,1).

The Fig 2 reveals a distinct trend: as the digital economy intensity (α) continues to increase, the firm’s stable evolution strategy converges towards 1 at a faster rate. It demonstrates that the digital economy intensity effectively enhances the synergy of GI&CV. Consequently, we can draw the following conclusion:

Conclusion: The digital economy fosters the synergy of GI&CV.

## 4. Metrological inspection

### 4.1 Research hypotheses

#### 4.1.1 The digital economy and the GI&CV synergy

The digital economy encompasses advancements in technological infrastructure and the extensive adoption of digital technology. This transition has fundamentally altered the production, distribution, and consumption patterns observed in the traditional economy. It has further facilitated the implementation of digital transformation strategies and organizational innovations [17], consequently presenting enterprises with an expanded array of innovation opportunities [43]. Particularly noteworthy are the opportunities arising in the domain of green technology and environmental preservation [44]. By leveraging digital technology, enterprises can effectively catalyze green innovation endeavors, such as energy conservation, emission reduction, and resource recycling [43, 45]. Moreover, the application of digital technology opens up broader market spaces and opportunities, thereby bolstering enterprises’ competitiveness and market value. Secondly, the digital economy has facilitated the swift dissemination of cognition and knowledge spillovers [46, 47]. This, in turn, enables enterprises to conveniently access and analyze data and information pertaining to environmental protection. Consequently, these enterprises are better equipped to devise scientifically rigorous and efficient green innovation strategies. By doing so, they stimulate the synergistic increase of company value. Thirdly, as the regional digital economy progresses, the public’s demand for sustainable development and environmental protection is steadily rising [48]. Concurrently, the market potential for green products and services is also expanding. Evidence of this trend can be found in the incorporation of digital economy in various countries’ important growth strategies. Many regions have implemented a range of supportive policies and measures, specifically aimed at bolstering green innovation and environmental conservation. The formulation of such policies and creation of supportive environments provide an auspicious external landscape for enterprises. These conditions incentivize and motivate enterprises to actively partake in green innovation initiatives, thereby enhancing their value and reputation. Hence, hypothesis 1 is proposed:

**Hypothesis 1 (H1):** The more the digital economy develops, the more it is conducive to synergistically improving GI&CV.

#### 4.1.2 Mediation effect of regional marketization level

Drawing upon the theory of information asymmetry, the digital economy has mitigated the information gap between firms and market stakeholders [49], facilitating expedited and seamless flow of market information. Market participants can more conveniently query, obtain, and share market information, improving the transparency and fairness of market information [50], thereby promoting the improvement of marketization level. Furthermore, digital marketing methods and online trading platforms enable enterprises to more accurately identify and meet market demands, improve market resource allocation efficiency, and reduce transaction costs [51, 52]. The improvement of regional marketization level intensifies competition among enterprises within the jurisdiction. Enterprise managers need to improve the environmental friendliness of products through innovation to satisfy differentiated market demands and gain long-term competitive advantage [44, 53], thereby stimulating the motivation for enterprises to engage in GI and enhance CV. Therefore, the hypothesis 2 is proposed:

**Hypothesis 2 (H2):** The digital economy promotes the synergistic improvement of GI&CV by enhancing the level of marketization.

#### 4.1.3 Mediation effect of digital transformation degree

Based on the resource-based theory, the digital economy offers robust digital infrastructure and favorable conditions for digital technology application [54], thereby presenting external environmental and resource advantages for firms’ digital transformation [55], empowering enterprises to effectively manage and leverage innovative resources. Through digital technology, enterprises can monitor and optimize the use of resources in real-time, reducing resource waste and unnecessary consumption [56]. This helps to improve resource utilization efficiency, reduce dependence on natural resources, and enhance the profitability and sustainability of enterprises from a long-term perspective [57], thereby facilitating the joint realization of GI&CV. Accordingly, the digital economy is conducive to building a cooperative, open and shared industrial value chain [58]. Enterprises have the opportunity to collaborate with various stakeholders within the value chain, including other enterprises, technology providers, innovators, and government departments, to foster collective efforts in driving digital transformation. Therefore, through digital platforms and technologies, enterprises can better engage in cross-border cooperation, share resources and knowledge, accelerate the revolution of green technologies, and enhance the value creation capabilities. This helps to promote the synergistic enhancement of GI&CV. Accordingly, the following hypothesis 3 is put forward:

**Hypothesis 3(H3):** The digital economy fosters the collaborative improvement of GI&CV by utilizing the degree of Digital transformation.

#### 4.1.4 The regulatory effect of environmental regulations

In response to the imperative need for green and sustainable development, environmental regulation stands as a fundamental governance measure for governments across different countries in their pursuit of sustainable economic and social development [59]. From a strategic perspective, the increase of government environmental regulation aggravate the shortage of capital market resources [60], encourage firms to invest in GI that can promote sustainable development, and improve the profitability of firms from a long-term perspective [61]. Thus, under the dual influence of environmental regulation and the digital economy, businesses can discern the advantages and risks inherent in the digital landscape. This understanding enables them to bolster environmental compliance, effectively harness external digital resources and tools, seamlessly integrate and manage the necessary elements for green innovation, and cultivate the development and application of green technologies, products, and services. These strategic initiatives not only cater to the diverse demands of the market but also foster consumer trust and loyalty. This will bring longer-term development and returns to the enterprise, thereby helping to synergistically enhance the GI and market value of the firms. Thus, the hypothesis 4 is proposed:

**Hypothesis 4 (H4):** Environmental regulations can help strengthen the fostering influence of digital economy on the synergy of GI&CV.

#### 4.1.5 The regulatory effect of green subsidies

Firms’ green innovation entails externalities and substantial risks [62]. Government green subsidies can offset the costs of GI, mitigate the risks associated with implementing green innovation activities, and thereby enhance enterprises’ willingness to engage in GI. Additionally, the subsidy policies of the government can also convey a positive message to the market, raising the expectations of social capital for green innovation in enterprises, guiding more social capital to invest in the field of GI, and alleviating the shortage of research and development funds during the process of GI [63]. In light of both green subsidies and the digital economy, there exists a synergistic effect on GI that can be effectively leveraged. This serves as an incentive for enterprises to augment their output of effective green innovation, thereby bolstering their capability for sustainable development and enhancing their market value. Consequently, we propose Hypothesis 5:

**Hypothesis 5(H5):** Green subsidies strengthen the fostering influence of digital economy on the synergy of GI&CV.

**Fig 3:**
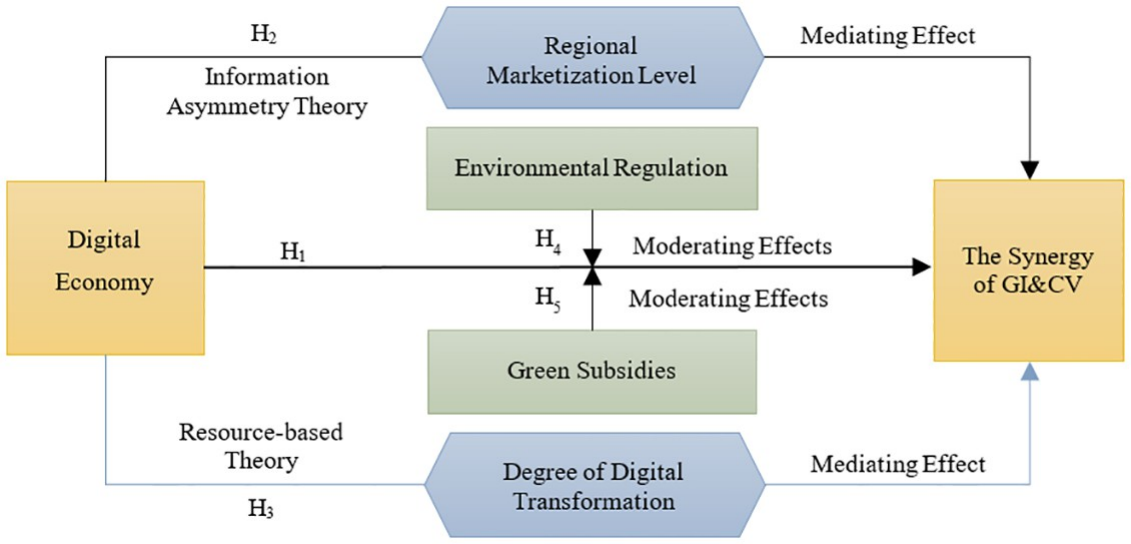
Research framework.

Accordingly, the framework of this study has been formed (Fig 3).

### 4.2 Research design

#### 4.2.1 Data sources

Considering the rapid development of China’s digital economy since approximately 2011, this study use a sample comprising Chinese listed firms’ data spanning from 2011 to 2020. The data of GI is sourced from the CNDRS database (https://www.cnrds.com). Data of the digital economy is gained by analyzing the *National Statistical Yearbook* and the *Statistical Yearbook* of each province. The marketization level is derived from the China Sub-Provincial Marketization Index Database (https://cmi.ssap.com.cn). CV, digital transformation and other controlled variables are sourced from the CSMAR database (https://data.csmar.com/). Financial firms, ST and *ST firms, were deleted from the samples to avoid the impact of abnormal data. Accordingly, 26,049 samples from 3767 listed companies were obtained.

#### 4.2.2 Variable definition

*(1) Explained variable*. Drawing upon the practices adopted by most scholars, the quantity of green patent authorizations is employed as a measure of firms’ GI. Additionally, considering that Tobin Q represents the ratio of a company’s market value to the replacement cost of its total assets, it is frequently utilized as an instrumental indicator to gauge company performance or market value (Hao et al., 2021) [64]. Therefore, Tobin Q is selected as a proxy variable for CV.

Furthermore, building upon the methodologies employed by Cheng et al. (2019) [65] and Dong et al. (2023) [66], this study proposes a capacity coupling coefficient model to evaluate the synergy of GI&CV. The details are outlined as follows:

Firstly, the maximum minimum method is applied to standardize GI&CV.

$${GI}_{i,n}=\frac{{GI}_{i,n}-min({GI}_{i,n})}{max\left({GI}_{i,n}\right)-min({GI}_{i,n})}$$ (9)

$${CV}_{i,n}=\frac{{CV}_{i,n}-min({CV}_{i,n})}{max\left({CV}_{i,n}\right)-min({CV}_{i,n})}$$ (10)

where the variable *GI*_*i*,*n*_ denotes the standardized GI of firm *i* in year *n*, while *CV*_*i*,*n*_ represents the standardized CV of firm *i* in year *n*.

Secondly, the calculation formula of the coupling degree *C*_*i*,*n*_ of the two subsystems is as follows:

$$C_{i.n}=\frac{2\sqrt[{2}]{{GI}_{i.n}\times {CV}_{i.n}}}{{GI}_{i.n}+{CV}_{i.n}}$$ (11)

Eq (11) demonstrates that a higher value of *C*_*i*, *n*_ indicates a stronger coupling between GI&CV, resulting in a more organized operation of the system. Conversely, a weaker coupling state between the systems leads to a more disordered development. Nevertheless, due to the dynamic changes and imbalanced nature of both GI&CV systems, there are still shortcomings in the calculation of the coupling degree mentioned above, which means that there may be errors in the measurement of overall functionality or comprehensive benefits during the dynamic development process [67], leading to the possibility of both positive and negative coupling between the two subsystems of GI&CV.

Therefore, in order to more reasonably measure the synergy of GI&CV, this study introduces the coupling coordination index (C*D*_*i*_) as an indicator to measure positive synergy, in order to solve the dynamic changes and non-equilibrium problems of subsystems. The equations are outlined as follows:

$${CD}_{i}=\sqrt[{2}]{C_{i}\times Q_{i}}$$ (12)

$$Q_{i}=aG_{i}+bS_{i}$$ (13)

where *CD*_*i*_ represents the synergy of GI&CV, while *Q*_*i*_ is an evaluation index used to reflect the development of various subsystems of GI&CV; *a* and *b* are the weight coefficients to be determined. Considering that GI&CV are equally important for the sustainable development of enterprises, the weight coefficients are assigned to 0.5 to calculate the synergy.

In order to distinguish the synergy between high-quality and low-quality GI&CV, the synergy between green invention patents and CV is adopted to represent the synergy of substantive GI&CV, while the synergy between green utility models patents and CV to represent the synergy of strategic GI&CV.

*(2) Explanatory variable*. Drawing on the study of Li and Wang (2022) [68] and Chen (2023) [69], five indicators are selected and measured by entropy weight method (Table 3).

**Table 3:** Measurement of the digital economy.

Primary indicators	Secondary Indicators	Calculation Basis	Attribute
Development level of digital economy	Internet penetration rate	Number of internet users per 100 people	+
	Number of Internet related practitioners	The proportion of computer service and software professionals in the total population of the region	+
	Internet related outputs	Total telecommunications business per capita	+
	Number of mobile internet users	Number of mobile phone users per 100 people	+
	Digital Financial inclusion index	Digital Financial inclusion index released by Peking University	+

*(3) Mediating variable*. Regional marketization level is measured using the marketization index published by the China Sub-Provincial Marketization Index Database (https://cmi.ssap.com.cn), which is a representative indicator widely used to gauge the relative degree of marketization in each region. In order to enhance the comparability of data from different years, this study conducts technical linkage processing on market-oriented indices, so that indices from different years can be vertically comparable across years.

Referring to the investigations of Verhoef et al. (2021) [70] and Feng et al. (2022) [17], the text analysis method is used to measure the degree of digital transformation (DT), that is, the number of times the text appears related to Big Data, artificial intelligence, cloud computing, Block Chain and digital technology applications in the annual reports of enterprises is summed up and logarithmically processed, and interpolate to fill in missing data. To distinguish the variations among different categories of digital transformation, we refer to the research conducted by Feng et al. (2022) [71] and ascertain that the first four categories constitute the underlying technical framework (UT), while the fifth category is defined as the technical practical application (TP).

*(4) Adjusting variables*. Following the research of Lanoie et al.(2008) [72], the logarithm of the ratio of industrial pollution control inputs to the value added of the industrial secondary sector in the current year in each region is used to measure the government’s environmental regulatory intensity.

Drawing on the study of Li et al. [73] and Hu et al. [74], items related to green development of enterprises from government fiscal incentives is selected to measure government green subsidies, including: special funds for the circular economy, energy conservation subsidies, subsidies for improving the quality of the air environment, subsidies for water conservation and emission reduction advanced enterprises, special subsidies for the cleaner production project, energy conservation advanced unit rewards, energy conservation rebates, environmental protection subsidy programs, energy-saving advanced unit rewards, energy-saving tax rebates, and environmental protection subsidy programs, etc. In order to eliminate the possible heteroskedasticity, the sum of green subsidies gained by firms is treated as a natural logarithm.

*(5) Control variables*. Based on the study of Meng et al. (2020) [75] and Bresciani et al. (2022) [76], scale (SCA), duration of establishment (TIM), financial leverage (LEV), growth ability (GRO), profitability (ROA), equity concentration (LCR), and proportion of independent directors (DLD) are selected as control variables. The main variables as shown in Table 4.

**Table 4:** Definition of main variables.

Types	Variables		Symbol	Interpretation
Explained variable	The synergy of GI&CV	The synergy of the total GI&CV	CD	Calculate using the capacity coupling coefficient model
		The synergy of substantive GI&CV	CD_S	Calculate using the capacity coupling coefficient model
		The synergy of strategic GI&CV	CD_C	Calculate using the capacity coupling coefficient model
Explanatory variable	Digital economy	Digital economy index	DE	The comprehensive index of digital economy calculated by entropy weight method
Mediating variables	Marketization level	Marketization index	MI	From the marketization index database
	Degree of digital transformation	Overall digital transformation	DT	Natural logarithm is obtained by adding 1 to the total number of text times of artificial intelligence, Big Data, cloud computing, Blockchain, and technical practical application
		Underlying technical architecture	UT	Natural logarithm is obtained by adding 1 to the total number of text times of artificial intelligence, Big Data, cloud computing and Blockchain
		Technical practical application	TP	Natural logarithm is obtained by adding 1 to the number of text times of technical practical application
Moderator variables	Environmental regulations intensity		ER	The Natural logarithm of the proportion of the industrial pollution control input of each province (city, district) in the added value of the industrial secondary sector of the economy
	Government green subsidies		BZ	Natural logarithm of the total amount of green subsidies received by the government every year
Control variables	Scale		SCA	The Natural logarithm of the total assets of the enterprise
	Duration of establishment		TIM	Establishment time of the enterprise
	Growth ability		GRO	Operating revenue growth/opening operating revenue
	Financial leverage		DEB	Total assets/total liabilities at the end of the period
	Profitability		ROA	Net profit/total assets
	Equity concentration		LCR	Shareholding ratio of the top 10 shareholders
	Proportion of independent directors		DLD	Proportion of independent directors to all directors

#### 4.2.3 Model construction

The findings from the Hausman tests conducted on the dataset indicate that a fixed effects model is well-suited for the analysis, allowing for the incorporation of dual fixed effects related to both time and industry. Furthermore, in order to mitigate any potential issues stemming from homoscedasticity, heteroscedasticity, and autocorrelation among variables, this study employs robust standard error clustered to province estimation.

Firstly, to investigate the influence of the digital economy on the synergy of GI&CV, the model (1) is constructed:

$${CD}_{i,t}=\alpha_{0}+\alpha_{1}{DE}_{t}+\alpha_{3}{Controls}_{i,t}+{year}_{t}+{industry}_{i}+\varepsilon_{i,t}$$ Model (1)

Secondly, the causal steps approach devised by Baron and Kenny(1986) [77] is adopted to verify the mediating effects of marketization level and degree of digital transformation. The model is as follows:

$${Mediator}_{i,t}=\beta_{0}+\beta_{1}{DE}_{t}+\beta_{2}{Controls}_{i,t}+{year}_{t}+{industry}_{i}+\varepsilon_{i,t}$$ Model (2)

$${CD}_{i,t}=\mu_{0}+\mu_{1}{Mediator}_{i,t}+\mu_{2}{DE}_{t}+\mu_{3}{Controls}_{i,t}+{year}_{t}+{industry}_{i}+\varepsilon_{i,t}$$ Model (3)

Thirdly, the model (4) is constructed to test the regulatory effects of environmental regulations and green subsidies:

$${CD}_{i,t}=\mu_{0}+\mu_{1}{Reg}_{i,t}\times {DE}_{t}+\mu_{2}{DE}_{t}+\mu_{3}{Reg}_{i,t}+\mu_{4}{Controls}_{i,t}+{year}_{t}+{industry}_{i}+\varepsilon_{i,t}$$ Model (4)

In model (1)–(4), *CD*_*i*,*t*_ represents the synergy of GI&CV, *DE*_*i*,*t*_ is denoted as the digital economy; *Mediator*_*i*,*t*_ is denoted as the mediator variables; *Controls*_*i*,*t*_ is denoted as all the control variables, *α*_*0*_, *β*_*0*_, *γ*_*0*_ and *μ*_*0*_ are the intercept terms, *α*_*1~n*_, *β*_*1~n*_, *γ*_*1~n*_ and *μ*_*1~n*_ are the correlation coefficients, and *ε*_*it*_ is the random error term.

#### 4.2.4 Descriptive statistics

The main continuous variables were double indented at the 1% quartile to avoid the effect of extreme values. Table 5 shows the descriptive statistics of the main variables.

**Table 5:** Descriptive statistics of the main variables.

Variables	N	Min	Max	Mean	SD
DE	26049	0.077	0.982	0.454	0.193
CD	26049	0.003	0.814	0.199	0.212
CD_S	26049	0.003	0.832	0.112	0.168
CD_C	26049	0.003	0.835	0.178	0.206
MI	26049	2.330	12	8.599	1.802
DT	26049	0	6.146	0.676	1.080
UT	26049	0	6.176	1.216	1.307
TP	26049	0	5.924	0.886	1.097
ER	26049	-0.110	4.124	2.635	0.761
BZ	25760	0	16.537	2.194	4.915
SCA	26049	17.64	28.19	22.12	1.268
AGE	26049	0.693	4.143	2.860	0.355
GRO	26049	-0.789	10.29	0.429	1.250
DEB	26049	0.0490	0.980	0.420	0.209
ROA	26049	-0.366	0.206	0.0350	0.0690
LCR	26049	1.523	4.627	4.049	0.291
DLD	26049	0.167	0.800	0.375	0.0550

### 4.3 Empirical results

#### 4.3.1 The influence of digital economy on the synergy of GI&CV

The findings displayed in column (1) of Table 6 reveal that the coefficient of the digital economy on the synergy of GI&CV is 0.1122, demonstrating statistical significance at the 1% level. This indicates that the digital economy plays a crucial role in encouraging firms to enhance the synergy of GI&CV. Further analysis, as shown in columns (2)-(3), confirms Hypothesis 1 by demonstrating that the growth of the digital economy significantly and positively affects both the substantive GI&CV synergy and the strategic GI&CV synergy.

**Table 6:** The influence of the digital economy on the synergy of GI&CV.

Variables	(1)	(2)	(3)
	Model (1)		
	CD	CD_S	CD_C
DE	0.1122***	0.1159***	0.0864***
	(0.0143)	(0.0124)	(0.0140)
SCA	0.0360***	0.0286***	0.0321***
	(0.0011)	(0.0010)	(0.0011)
AGE	-0.0442***	-0.0234***	-0.0402***
	(0.0040)	(0.0033)	(0.0039)
GRO	0.0029**	0.0031***	0.0020*
	(0.0009)	(0.0007)	(0.0009)
DEB	0.0568***	0.0089	0.0695***
	(0.0078)	(0.0062)	(0.0076)
ROA	0.1018***	0.0244	0.1019***
	(0.0203)	(0.0162)	(0.0197)
LCR	-0.0495***	-0.0407***	-0.0366***
	(0.0046)	(0.0039)	(0.0045)
DLD	-0.0705**	-0.0081	-0.0731***
	(0.0218)	(0.0181)	(0.0214)
Cons	-0.3848***	-0.3462***	-0.3875***
	(0.0322)	(0.0277)	(0.0316)
Time FE	YES	YES	YES
Industry FE	YES	YES	YES
*N*	26049	26049	26049
*R* ^2^	0.142	0.083	0.131

Note: Robust standard errors clustered to province in parentheses,

* p<0.05,

** p<0.01,

*** p<0.001.

#### 4.3.2 Robustness testing

*(1) Robustness testing by replacing the dependent variable*. In comparison to the authorized green patents, the green patent applications offers a more timely reflection of innovation. Therefore, for the purpose of robustness testing, this study substitutes the count of authorized green patents with the count of green patent applications (GIA). The empirical results of the substituted dependent variable are presented in columns (1)-(3) of Table 7 for the robustness test. The statistical significance of the digital economy’s coefficient on the synergy between GI&CV at the 1% level provides clear evidence, affirming the robustness of the findings articulated in this study.

**Table 7:** Empirical results of replacing the dependent variable.

Variables	(1)	(2)	(3)
	Model(1)		
	CD_Q	CDS_Q	CDC_Q
DE	0.1140***	0.1254***	0.0788***
	(0.0149)	(0.0145)	(0.0142)
SCA	0.0405***	0.0401***	0.0339***
	(0.0012)	(0.0011)	(0.0011)
AGE	-0.0452***	-0.0373***	-0.0418***
	(0.0041)	(0.0039)	(0.0040)
GRO	0.0037***	0.0045***	0.0022*
	(0.0009)	(0.0009)	(0.0009)
DEB	0.0495***	0.0265***	0.0712***
	(0.0081)	(0.0075)	(0.0078)
ROA	0.1773***	0.1386***	0.1342***
	(0.0215)	(0.0200)	(0.0202)
LCR	-0.0580***	-0.0515***	-0.0403***
	(0.0046)	(0.0044)	(0.0045)
DLD	-0.0550*	-0.0249	-0.0714**
	(0.0229)	(0.0217)	(0.0218)
Cons	-0.4230***	-0.4898***	-0.4053***
	(0.0334)	(0.0317)	(0.0322)
Time FE	YES	YES	YES
Industry FE	YES	YES	YES
*N*	26049	26023	25986
*R* ^2^	0.150	0.125	0.140

Note: Robust standard errors clustered to province in parentheses,

* p<0.05,

** p<0.01,

*** p<0.001.

*(2) Robustness testing by replacing the explanatory variable*. From the perspective of local government digital economic policies, this study acquires original documents of government work reports from regional government websites. Subsequently, frequency analysis is conducted on the textual content of these reports to quantify the frequency of relevant terms, which serves as a proxy variable for the degree of digital economic policy support across different regions. Additionally, to mitigate potential heteroscedasticity in the data, logarithmic transformation is applied. By analyzing the empirical findings presented in Table 8, it becomes evident that the advancement of the digital economy continues to significantly facilitate the enhancement of the synergy of GI&CV, thereby affirming the robustness of the principal findings.

**Table 8:** Robustness testing by replacing the explanatory variable.

Variables	(1)	(2)	(3)
	Model (1)		
	CD	CD_S	CD_C
DE_2	0.0441***	0.0331***	0.0396***
	(0.0063)	(0.0051)	(0.0063)
SCA	0.0363***	0.0290***	0.0324***
	(0.0011)	(0.0010)	(0.0011)
AGE	-0.0461***	-0.0253***	-0.0417***
	(0.0040)	(0.0033)	(0.0039)
GRO	0.0032***	0.0034***	0.0023**
	(0.0009)	(0.0007)	(0.0009)
DEB	0.0523***	0.0041	0.0661***
	(0.0078)	(0.0062)	(0.0075)
ROA	0.1009***	0.0232	0.1013***
	(0.0203)	(0.0163)	(0.0196)
LCR	-0.0478***	-0.0389***	-0.0353***
	(0.0046)	(0.0039)	(0.0045)
DLD	-0.0716**	-0.0079	-0.0745***
	(0.0218)	(0.0181)	(0.0213)
Cons	-0.7827***	-0.6432***	-0.7449***
	(0.0658)	(0.0538)	(0.0649)
Time FE	YES	YES	YES
Industry FE	YES	YES	YES
*N*	26049	26049	26049
*R* ^2^	0.142	0.081	0.131

Note: Robust standard errors clustered to province in parentheses,

* p<0.05,

** p<0.01,

*** p<0.001.

*(3) Robustness testing by using one period lag of explanatory variables*. To ascertain the robustness of the findings, a robustness test is conducted by employing a one-period lag of the explanatory variables. The empirical results presented in Table 9 provide evidence that the progress of the digital economy continues to exert a substantial positive impact on the synergistic advancement of GI&CV. These findings serve to validate the principal conclusion once more.

**Table 9:** Robustness test by using one period lag of explanatory variables.

Variables	(1)	(2)	(3)
	Model (1)		
	CD_1	CD_S1	CD_C1
DE	0.1223***	0.1246***	0.0969***
	(0.0168)	(0.0146)	(0.0164)
SCA	0.0332***	0.0279***	0.0291***
	(0.0013)	(0.0011)	(0.0013)
AGE	-0.0438***	-0.0253***	-0.0402***
	(0.0044)	(0.0037)	(0.0043)
GRO	0.0031**	0.0036***	0.0020*
	(0.0010)	(0.0008)	(0.0009)
DEB	0.0547***	0.0011	0.0725***
	(0.0089)	(0.0071)	(0.0086)
ROA	0.0914***	0.0143	0.1016***
	(0.0258)	(0.0210)	(0.0247)
LCR	-0.0378***	-0.0358***	-0.0254***
	(0.0050)	(0.0043)	(0.0049)
DLD	-0.0765**	0.0068	-0.0906***
	(0.0246)	(0.0206)	(0.0240)
Cons	-0.3523***	-0.3310***	-0.3559***
	(0.0358)	(0.0310)	(0.0350)
Time FE	YES	YES	YES
Industry FE	YES	YES	YES
*N*	21453	21453	21453
*R* ^2^	0.138	0.080	0.128

Note: Robust standard errors clustered to province in parentheses,

* p<0.05,

** p<0.01,

*** p<0.001.

#### 4.3.3 Endogeneity test

In order to mitigate potential endogeneity issues that could introduce bias in the empirical findings, the model is re-estimated utilizing the instrumental variable method and 2SLS estimation. Building on the investigation exerted by Wu et al. (2022), internet penetration is employed as the instrumental variable. The findings in Table 10 indicate that the initial hypothesis of inadequate identification of the instrumental variable and the original hypothesis of a weak instrumental variable are all rejected, providing further support for the validity of the chosen instrumental variables. The inclusion of instrumental variables in the analysis sustains the presence of a substantial positive relationship between the digital economy and the synergy of GI&CV for firms in the second stage. This reinforces the consistency and robustness of the key findings.

**Table 10:** Endogeneity test.

Variables	(1)	(2)	(3)	(4)
	2SLS			
	Phase 1	Phase 2		
	DE	CD	CD_S	CD_C
IV	0.0057***			
	(0.0000)			
DE		0.1934***	0.1425***	0.1835***
		(0.0189)	(0.0159)	(0.0185)
SCA	0.0016***	0.0357***	0.0285***	0.0318***
	(0.0004)	(0.0011)	(0.0010)	(0.0011)
AGE	-0.0085***	-0.0428***	-0.0229***	-0.0386***
	(0.0010)	(0.0040)	(0.0033)	(0.0039)
GRO	0.0012***	0.0028***	0.0031***	0.0019**
	(0.0003)	(0.0009)	(0.0007)	(0.0009)
DEB	-0.0119***	0.0603***	0.0100	0.0738***
	(0.0022)	(0.0078)	(0.0062)	(0.0076)
ROA	-0.0123*	0.1032***	0.0249	0.1036***
	(0.0066)	(0.0203)	(0.0162)	(0.0197)
LCR	0.0026**	-0.0508***	-0.0412***	-0.0382***
	(0.0012)	(0.0046)	(0.0039)	(0.0045)
DLD	-0.0094	-0.0732***	-0.0090	-0.0763***
	(0.0062)	(0.0218)	0.0285***	(0.0214)
Cons	-0.1320***	-0.3881***	-0.3473***	-0.3914***
	(0.0096)	(0.0322)	(0.0276)	(0.0316)
Time FE	YES	YES	YES	YES
Industry FE	YES	YES	YES	YES
*N*	26,049	26,049	26,049	26,049
*R* ^2^		0.142	0.084	0.130
Kleibergen-Paap rk LM statistic		9991.365***		
Cragg-Donald Wald F statistic		37631.66		
Stock-Yogo weak ID test critical values		16.38		

Note: Robust standard errors clustered to province in parentheses,

* p<0.05,

** p<0.01,

*** p<0.001.

#### 4.3.4 Transmission mechanism of the influence of digital economy on the synergy of GI&CV

*(1) The mediating effect of regional marketization level*. The empirical results pertaining to the mediating role of regional marketization between the digital economy and the synergistic effects of GI&CV are presented in Table 11. It is noteworthy that the influence of digital economy on marketization level, as well as the influence of marketization on GI&CV synergy, are both found to be significantly positive. These findings provide evidence that the promotion of GI&CV synergy is facilitated by the advancement of digital economy, through the enhancement of marketization level. Thus, Hypothesis 2 is validated.

**Table 11:** Mediating effects test for regional marketization levels.

Variables	(1)	(2)	(3)	(4)
	Model (2)	Model (3)	Model (3)	Model (3)
	MI	CD	CD_S	CD_C
MI		0.0088***	0.0035***	0.0094***
		(0.0010)	(0.0008)	(0.0010)
DE	12.4055***	0.0029	0.0721***	-0.0301
	(0.1076)	(0.0191)	(0.0169)	(0.0185)
SCA	-0.1171***	0.0370***	0.0290***	0.0332***
	(0.0075)	(0.0011)	(0.0010)	(0.0011)
AGE	-0.0733**	-0.0436***	-0.0231***	-0.0396***
	(0.0223)	(0.0040)	(0.0033)	(0.0039)
GRO	-0.0626***	0.0035***	0.0033***	0.0026**
	(0.0064)	(0.0009)	(0.0007)	(0.0009)
DEB	0.1346**	0.0556***	0.0084	0.0683***
	(0.0495)	(0.0078)	(0.0062)	(0.0076)
ROA	0.9445***	0.0935***	0.0211	0.0930***
	(0.1298)	(0.0202)	(0.0162)	(0.0196)
LCR	0.2768***	-0.0519***	-0.0417***	-0.0392***
	(0.0284)	(0.0046)	(0.0039)	(0.0045)
DLD	-0.1156	-0.0694**	-0.0077	-0.0720***
	(0.1337)	(0.0218)	(0.0181)	(0.0213)
Cons	6.0199***	-0.4379***	-0.3675***	-0.4440***
	(0.2101)	(0.0327)	(0.0280)	(0.0321)
Time FE	YES	YES	YES	YES
Industry FE	YES	YES	YES	YES
*N*	26049	26049	26049	26049
*R* ^2^	0.571	0.144	0.084	0.134

Note: Robust standard errors clustered to province in parentheses,

* p<0.05,

** p<0.01,

*** p<0.001.

*(2) The mediating effect of the degree of digital transformation*. The findings in Table 12 indicates that the coefficients of the degree of digital economy development on DT, UT, and TP are all significantly positive, and the coefficients of the DT, UT, and TP on the synergy of GI&CV are all significantly dominant. It can be observed that the influence coefficient of digital economic on the synergy of GI&CV decreases after adding the mediator variables, which demonstrates that the degree of digital transformation plays a partially mediating effect between digital economic and the synergy of GI&CV, and Hypothesis 3 is verified.

**Table 12:** Mediating effects of the degree of digital transformation.

Variables	(1)	(2)	(3)	(4)	(5)	(6)
	Model(2)	Model(3)	Model(2)	Model(3)	Model(2)	Model(3)
	DT	CD	UT	CD	TP	CD
DT		0.0256***				
		(0.0012)				
UT				0.0400***		
				(0.0014)		
TP						0.0147***
						(0.0013)
DE	1.3448***	0.0777***	1.2374***	0.0627***	0.7632***	0.1009***
	(0.0815)	(0.0143)	(0.0717)	(0.0141)	(0.0725)	(0.0143)
SCA	0.0923***	0.0336***	0.0387***	0.0344***	0.0858***	0.0347***
	(0.0063)	(0.0011)	(0.0052)	(0.0011)	(0.0057)	(0.0011)
AGE	-0.2638***	-0.0374***	-0.1906***	-0.0366***	-0.1714***	-0.0417***
	(0.0222)	(0.0040)	(0.0188)	(0.0040)	(0.0195)	(0.0040)
GRO	0.0202***	0.0024**	0.0322***	0.0016	0.0045	0.0028**
	(0.0053)	(0.0009)	(0.0043)	(0.0009)	(0.0045)	(0.0009)
DEB	-0.1084*	0.0596***	-0.0286	0.0579***	-0.0718	0.0579***
	(0.0424)	(0.0077)	(0.0349)	(0.0076)	(0.0384)	(0.0078)
ROA	-0.0675	0.1035***	0.0119	0.1013***	-0.1026	0.1033***
	(0.1188)	(0.0199)	(0.1047)	(0.0198)	(0.1081)	(0.0202)
LCR	-0.0843***	-0.0473***	-0.1856***	-0.0421***	-0.0037	-0.0494***
	(0.0242)	(0.0045)	(0.0206)	(0.0045)	(0.0214)	(0.0046)
DLD	0.8145***	-0.0913***	0.5360***	-0.0919***	0.5745***	-0.0789***
	(0.1247)	(0.0216)	(0.1045)	(0.0213)	(0.1135)	(0.0218)
Cons	-1.3376***	-0.3505***	-0.1425	-0.3791***	-1.5842***	-0.3615***
	(0.1720)	(0.0321)	(0.1404)	(0.0318)	(0.1540)	(0.0323)
Time FE	YES	YES	YES	YES	YES	YES
Industry FE	YES	YES	YES	YES	YES	YES
*N*	26049	26049	26049	26049	26049	26049
*R* ^2^	0.346	0.158	0.336	0.169	0.257	0.146

Note: Robust standard errors clustered to province in parentheses,

* p<0.05,

** p<0.01,

*** p<0.001.

#### 4.3.5 The Moderating effects of environmental regulation and green subsidies

The empirical results in Table 13 reveal that the coefficient of the cross-multiplier term of environmental regulation and digital economy on the synergy of GI&CV, as well as the cross-multiplier term of green subsidies and the digital economy, are both found to be significantly positive, which indicates that environmental regulation and green subsidies strengthens the impact of the digital economy on the synergy of GI&CV, and Hypothesis 4 and 5 are verified.

**Table 13:** Empirical tests of moderating effects.

Variables	(1)	(2)	(3)	(4)	(5)	(6)
	Model (2)	Model (3)	Model (2)	Model (3)	Model (2)	Model (3)
	CD	CD_S	CD_C	CD	CD_S	CD_C
DE×ER	0.0396***	0.0180**	0.0427***			
	(0.0066)	(0.0057)	(0.0064)			
DE×BZ				0.0072***	0.0035*	0.0085***
				(0.0019)	(0.0017)	(0.0018)
ER	-0.0136***	-0.0115***	-0.0123***			
	(0.0020)	(0.0017)	(0.0020)			
BZ				0.0011**	0.0008*	0.0009*
				(0.0004)	(0.0003)	(0.0004)
DE	0.1058***	0.0988***	0.0860***	0.1136***	0.1153***	0.0889***
	(0.0154)	(0.0131)	(0.0151)	(0.0145)	(0.0126)	(0.0142)
SCA	0.0364***	0.0289***	0.0326***	0.0357***	0.0280***	0.0320***
	(0.0011)	(0.0010)	(0.0011)	(0.0012)	(0.0010)	(0.0011)
AGE	-0.0442***	-0.0233***	-0.0402***	-0.0437***	-0.0224***	-0.0396***
	(0.0040)	(0.0033)	(0.0039)	(0.0041)	(0.0033)	(0.0040)
GRO	0.0030**	0.0031***	0.0021*	0.0030**	0.0032***	0.0020*
	(0.0009)	(0.0007)	(0.0009)	(0.0009)	(0.0007)	(0.0009)
DEB	0.0572***	0.0091	0.0699***	0.0578***	0.0086	0.0715***
	(0.0078)	(0.0062)	(0.0076)	(0.0078)	(0.0062)	(0.0076)
ROA	0.0993***	0.0244	0.0987***	0.0995***	0.0230	0.0987***
	(0.0203)	(0.0162)	(0.0197)	(0.0203)	(0.0162)	(0.0197)
LCR	-0.0509***	-0.0414***	-0.0380***	-0.0499***	-0.0398***	-0.0369***
	(0.0046)	(0.0039)	(0.0045)	(0.0046)	(0.0039)	(0.0045)
DLD	-0.0742***	-0.0113	-0.0765***	-0.0740***	-0.0132	-0.0778***
	(0.0218)	(0.0180)	(0.0213)	(0.0220)	(0.0181)	(0.0215)
Cons	-0.3521***	-0.3169***	-0.3587***	-0.3791***	-1.5842***	-0.3615***
	(0.0327)	(0.0280)	(0.0320)	(0.0318)	(0.1540)	(0.0323)
Time FE	YES	YES	YES	YES	YES	YES
Industry FE	YES	YES	YES	YES	YES	YES
*N*	26049	26049	26049	25760	25760	25760
*R* ^2^	0.144	0.085	0.133	0.141	0.081	0.131

Note: Robust standard errors clustered to province in parentheses,

* p<0.05,

** p<0.01,

*** p<0.001.

#### 4.3.6 Heterogeneity analysis

By conducting empirical tests on groups, we are able to examine the variations in how the digital economy influences the synergy between GI&CV across different firms. This approach enables us to offer precise and practical recommendations to the government on how to foster the collaborative growth of GI&CV.

*(1) Grouping by economic regions*. The findings presented in Table 14 indicate a significantly higher level of significance in the eastern region compared to the central and northeastern regions. This observation suggests that the impact of the digital economy on the synergy between GI&CV is more pronounced in the eastern region, highlighting a greater sensitivity to this relationship in that specific geographic area. Nevertheless, the correlation coefficients for firms in the western and northeastern regions are greater in magnitude compared to eastern region. This observation implies that the central and northeastern regions experience a larger increase in GI&CV synergy for every unit increase in digital economy.

**Table 14:** Grouping according to economic regions.

Variables	(1)	(2)	(3)	(4)
	Model (1)			
	Eastern region	Central Region	Western region	Northeast region
	CD	CD	CD	CD
DE	0.0727***	0.5336*	0.3178	0.5728*
	(0.0192)	(0.2223)	(0.1726)	(0.2510)
SCA	0.0343***	0.0429***	0.0382***	0.0311***
	(0.0014)	(0.0031)	(0.0034)	(0.0045)
AGE	-0.0334***	-0.0514***	-0.0967***	-0.0194
	(0.0048)	(0.0119)	(0.0129)	(0.0167)
GRO	0.0035**	0.0080**	0.0055*	-0.0055**
	(0.0012)	(0.0028)	(0.0027)	(0.0020)
DEB	0.0780***	0.0313	0.0658**	-0.1218***
	(0.0096)	(0.0212)	(0.0209)	(0.0298)
ROA	0.1023***	0.1165*	0.0738	-0.0106
	(0.0244)	(0.0583)	(0.0538)	(0.0879)
LCR	-0.0547***	-0.0477***	-0.0323**	-0.0452*
	(0.0057)	(0.0130)	(0.0119)	(0.0175)
DLD	-0.0798**	-0.0151	-0.0111	-0.0916
	(0.0265)	(0.0655)	(0.0606)	(0.0790)
Cons	-0.3598***	-0.5495***	-0.4046***	-0.3525**
	(0.0404)	(0.0848)	(0.0930)	(0.1216)
Time FE	YES	YES	YES	YES
Industry FE	YES	YES	YES	YES
*N*	18007	3674	3100	1268
*R* ^2^	0.152	0.161	0.132	0.118

Note: Robust standard errors clustered to province in parentheses,

* p<0.05,

** p<0.01,

*** p<0.001.

Moreover, it is worth noting that the coefficient representing the influence of digital economic on the synergy of GI&CV located in the western region is deemed insignificant, as indicated by the empirical results. This insignificance suggests that digital economic development does not enhance the synergy of GI&CV among enterprises in the western region.

*(2) Grouping by heavy and non-heavy polluting industries*. The findings in Table 15 reveal that the influence of digital economy on both synergy of GI&CV in non-heavily polluting industries is significantly positive at 1% level; whereas the influence of digital economy is significant only on synergy of substantive GI&CV in the group of heavily polluting industries. This indicates that the digital economy is more effective in fostering the synergy of GI&CV in non-heavily polluting industries.

**Table 15:** Grouping by heavy and non-heavy polluting industries.

Variables	(1)	(2)	(3)	(4)	(5)	(6)
	Model (1)					
	Heavy polluting industries group			Non-heavy polluting industries group		
	CD	CD_S	CD_C	CD	CD_S	CD_C
DE	0.0266	0.0676**	0.0405	0.0993***	0.1089***	0.0605***
	(0.0290)	(0.0252)	(0.0283)	(0.0165)	(0.0143)	(0.0161)
SCA	0.0453***	0.0323***	0.0417***	0.0342***	0.0284***	0.0303***
	(0.0020)	(0.0017)	(0.0019)	(0.0014)	(0.0012)	(0.0014)
AGE	-0.0512***	-0.0277***	-0.0418***	-0.0375***	-0.0198***	-0.0349***
	(0.0080)	(0.0062)	(0.0077)	(0.0046)	(0.0039)	(0.0045)
GRO	-0.0035	-0.0012	-0.0029	0.0032**	0.0035***	0.0018
	(0.0019)	(0.0014)	(0.0018)	(0.0010)	(0.0008)	(0.0010)
DEB	0.0037	-0.0238*	0.0097	0.0860***	0.0247***	0.1022***
	(0.0146)	(0.0116)	(0.0137)	(0.0092)	(0.0074)	(0.0090)
ROA	0.1228**	0.0021	0.1230**	0.0896***	0.0243	0.0891***
	(0.0428)	(0.0319)	(0.0407)	(0.0229)	(0.0189)	(0.0223)
LCR	-0.0370***	-0.0382***	-0.0206*	-0.0585***	-0.0438***	-0.0469***
	(0.0084)	(0.0070)	(0.0080)	(0.0054)	(0.0046)	(0.0053)
DLD	-0.2017***	-0.0540	-0.1920***	-0.0428	-0.0032	-0.0498*
	(0.0410)	(0.0331)	(0.0394)	(0.0254)	(0.0213)	(0.0249)
Cons	-0.5105***	-0.3806***	-0.5476***	-0.3439***	-0.3479***	-0.3313***
	(0.0575)	(0.0502)	(0.0561)	(0.0385)	(0.0331)	(0.0379)
Time FE	YES	YES	YES	YES	YES	YES
Industry FE	YES	YES	YES	YES	YES	YES
*N*	7000	7000	7000	18945	18945	18945
*R* ^2^	0.106	0.064	0.109	0.175	0.099	0.162

Note: Robust standard errors clustered to province in parentheses,

* p<0.05,

** p<0.01,

*** p<0.001.

## 5. Discussion

The main effects test results indicate that The digital economy plays a pivotal role in driving the synergistic improvement of GI&CV, as evidenced by research conducted by Peng et al. (2023) [3], Huang et al. (2023) [78], and Wang et al. (2023) [79]. With the advancement of the digital economy, firms can effectively monitor and manage the allocation and utilization of green innovation resources, leading to optimal allocation and reduced negative environmental externalities. Furthermore, this facilitates the creation of sustainable profit sources for enterprises, thereby promoting the synergistic enhancement of GI&CV.

The results of the mediation analysis demonstrate that the digital economy facilitates the promotion of GI&CV synergies by augmenting marketization levels, which is similar to the research of Zhang et al.(2023) [80] and Peng et al.(2022) [81]. Drawing upon information asymmetry theory, the progress of the digital economy mitigates information asymmetry, enhancing the transparency and fairness of market information. Consequently, marketization levels are elevated, as intensified market-based competition compels enterprises to seek long-term competitive advantages through the joint enhancement of GI&CV. Thus, the level of marketization serves as a pivotal intermediary variable in the promotion of GI&CV synergistic enhancement facilitated by digital economy development. Furthermore, digital transformation serves as another crucial mediating factor in the synergies between firms’ GI&CV influenced by the evolution of the digital economy, as corroborated by research conducted by Xu et al. (2023) [14] and Wang et al. (2024) [82]. Based on the resource-based theory, digital economy provides an external environment and resource advantages for firms’ digital transformation. These firms can better cooperate across borders, share resources and knowledge, accelerate the revolution of green technology and enhance the enterprise value creation ability. This study also found that Environmental regulations and green subsidies exert a positive moderating effect between digital economy development and firms’ GI&CV synergies, which is supported by the study of. Liu et al.(2020) [83]. This demonstrates the synergistic effect of government constraints and incentive policies in facilitating the enhancement of GI&CV synergies.

The results of the heterogeneity analysis reveal that the influence of digital economy on the synergistic degree of GI&CV in the eastern area is most incentive, which is similar to the research of Li et al. (2022) [16] and Chen et al. (2023) [84]. In the context of the digital economy, higher marketization and resource agglomeration of multiple innovation factors are more conducive to driving the synergistic development of GI&CV of enterprises in the eastern area. Furthermore, the advancement of the digital economy contributes to a significant synergistic enhancement of firms’ GI&CV in the western and northeastern areas, aligning with the findings of a study by Huang et al. (2023) [78]. This phenomenon can be attributed to the implementation of China’s industrial restructuring and the Northeast Revitalization Strategy in recent years, prompting enterprises in these regions to place greater emphasis on the interconnected influence of digital economic on their own environmentally sustainable and high-quality growth. Consequently, every unit of progress in digital economic development yields a higher increment in the synergies of GI&CV among enterprises in the eastern and central areas. It’s worth noting that the influence of digital economic on the synergy of GI&CV in the western region lacks significance, as demonstrated by the research conducted by Wang et al. (2024) [85]. This discrepancy may arise from the western region predominantly consisting of underdeveloped areas, characterized by low levels of digital economic development, inadequate digital infrastructure, and limited benefits derived from digital industry agglomeration. Additionally, digital economy plays a more significant role in facilitating the synergy of GI&CV for non-heavily polluting firms. These firms offer products and services that are more aligned with green development objectives. Consequently, they can effectively leverage the benefits of digital economic development to optimize the allocation of green innovation resources among firms. This optimization process contributes to the enhancement of the synergy of GI&CV, thereby promoting the realization of mutually beneficial outcomes.

## 6. Conclusions and recommendations

### 6.1 Conclusions

This study utilizes an evolutionary game model to elucidate the mechanism of the digital economy on the synergies between GI&CV. Subsequently, an empirical analysis is conducted using data from Chinese publicly traded companies spanning from 2011 to 2020 to verify the impact of the digital economy on the synergy between GI&CV. The main conclusions are as follows: Firstly, digital economy significantly fosters the improvement of firms’ GI&CV synergies. This result is validated in both evolutionary game analysis and several robust empirical tests. Secondly, in terms of the transmission mechanism, the regional marketization level and the digital transformation of firms play essential roles in the transmission between digital economy and synergy of GI&CV. Thirdly, the results of the expansiveness analysis indicate that environmental regulation and green subsidies play cooperative effects with digital economy on the synergy of firms’ GI&CV. Fourthly, digital economic presents the most sensitive impact on the synergistic degree of GI&CV of firms in the eastern area, and the synergistic enhancement effect on GI&CV of firms in the western and northeastern areas is more obvious, and that digital economic is more effective in promoting the synergistic degree of GI&CV of non-heavily polluting firms.

### 6.2 Recommendations

#### 6.2.1 Countermeasures for the government

Firstly, it is recommended that the government prioritize strengthening information infrastructure to cultivate the expansion of the digital economy. This entails enhancing the seamless integration of information resources and facilitating the opening of the information "artery," which plays a vital role in promoting firms’ green sustainable development and CV.

Secondly, the government should formulate and improve the regulatory policies and regulations related to the digital economy to ensure fair competition in the market and information security. The relevant departments can establish a monitoring and evaluation mechanism for digital economy construction, adjust policies and measures timely, and promote the growth of digital economy.

Thirdly, the government should establish an effective market-based GI ecosystem and continuously upgrade the regional marketization level in order to enhance the synergy of the digital economy development on enterprise GI&CV.

Fourth, the government should further enhance environmental constraints and incentive policies to strengthen fostering effect the digital economy on the synergy GI&CV by upgrading environmental regulations and green subsidies.

#### 6.2.2 Countermeasures for the enterprises

Firstly, enterprises ought to harness the technological advancements provided by the digital economy such as Internet of Things and big data analysis to gain deeper insights into their own operations and market demands. By utilizing data analysis, enterprises can enhance their ability to identify potential green innovation opportunities, thereby achieving a synergistic enhancement of their GI&CV.

Secondly, enterprises should capitalize on the digital economy’s opportunities to facilitate their digital transformation. This includes improving the efficiency of transforming their GI resources, and introducing more environmentally friendly and sustainable products and services. These actions will contribute to fostering the synergy between GI&CV.

Thirdly, enterprises operating in the eastern, central, and western areas, as well as those classified as non-heavily polluting, should absorb the chances presented by the digital economy to enhance the synergistic effects of GI&CV. Such endeavors will not only strengthen their market competitiveness but also pave the way for sustainable development.

## Supporting information

S1 Data (XLSX)
